# Peroxisome Proliferator-Activated Receptor-*γ* in Amyotrophic Lateral Sclerosis and Huntington's Disease

**DOI:** 10.1155/2008/418765

**Published:** 2008-04-24

**Authors:** Mahmoud Kiaei

**Affiliations:** Department of Neurology and Neuroscience, Weill Medical College of Cornell University, New York-Presbyterian Hospital, New York, NY 10065, USA

## Abstract

Amyotrophic lateral sclerosis (ALS) is a debilitating and one of the most common adult-onset neurodegenerative diseases with the prevalence of about 5 per 100 000 individuals. It results in the progressive loss of upper and lower motor neurons and leads to gradual muscle weakening ultimately causing paralysis and death. ALS has an obscure cause and currently no effective treatment exists. In this review, a potentially important pathway is described that can be activated by peroxisome proliferator-activated receptor-*γ* (PPAR-*γ*) agonists and has the ability to block the neuropathological damage caused by inflammation in ALS and possibly in other neudegenerative diseases like Huntington's disease (HD). Neuroinflammation is a common pathological feature in neurodegenerative diseases. Therefore, PPAR-*γ* agonists are thought to be neuroprotective in ALS and HD. We and others have tested the neuroprotective effect of pioglitazone (Actos), a PPAR-*γ* agonist, in G93A SOD1 transgenic mouse model of ALS and found significant increase in survival of G93A SOD1 mice. These findings suggest that PPAR-*γ* may be an important regulator of neuroinflammation and possibly a new target for the development of therapeutic strategies for ALS. The involvement of PPAR-*γ* in HD is currently under investigation, one study finds that the treatment with rosiglitazone had no protection in R6/2 transgenic mouse model of HD. PPAR-*γ* coactivator-1*α* (PGC-1*α*) is a transcriptional coactivator that works together with combination of other transcription factors like PPAR-*γ* in the regulation of mitochondrial biogenesis. Therefore, PPAR-*γ* is a possible target for ALS and HD as it functions as transcription factor that interacts with PGC-1*α*. In this review, the role of PPAR-*γ* in ALS and HD is discussed based on the current literature and hypotheses.

## 1. INTRODUCTION

Peroxisome proliferator-activated receptors (PPARs) are ligand-activated transcription factors that belong to the nuclear hormone receptor superfamily which includes PPAR-*γ*, PPAR-*α*, and PPAR-*β*/*δ*. PPAR-*γ* is the most studied receptor and has two isoforms produced due to alternative splicing and alternate translation initiation: PPAR-*γ*_1_ and PPAR-*γ*_2_ [1, 2]. Another ligand-activated transcription factor is retinoid-X receptor from the same superfamily that forms heterodimeric complexes with PPARs in response to ligand binding. These heterodimeric complexes bind to the cis-acting sequences that are also called peroxisome proliferators response element (PPRE) on DNA to activate or inactivate the transcription of target genes (for further details see [3–5]).

PPARs are ligand-dependent transcription factors that bind to specific PPREs and enhance the expression of regulated genes [6]. PPARs regulate the expression of target genes, in particular those associated with lipid metabolism [7–9]. PPAR isotypes appear to exhibit distinct patterns of tissue distribution and differ considerably in their ligand binding domains, implying that they possibly perform different functions in different cell types [2, 10–13]. PPAR-*α* is expressed in high levels in hepatocytes, entrocytes, and kidney [12]. PPAR-*α* is implicated to be responsible for the peroxisome proliferator-induced pleiotropic responses [14]. PPAR-*α* and *δ* appear primarily to stimulate oxidative lipid metabolism, while PPAR-*γ* is principally involved in the cellular assimilation of lipids via anabolic pathways. Recently, other functions for PPAR-*γ* are described, such as neuroprotection in ischemia [15] and its effect on spinal cord injury (SCI) [16] (also for review see [17]).

**Figure 1:**
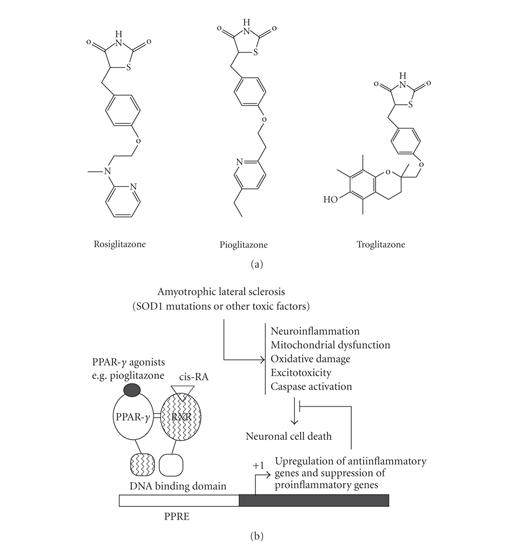
(a) Structure of PPAR agonists, (b) schematic diagrams linking mechanisms of neuronal cell death in ALS and a representation of PPAR-*γ* activation. The mechanisms and pathways implicated in the pathogenesis of ALS that lead to the demise of motor neurons are multiple. Activation of PPAR-*γ* by pioglitazone has the potential to block inflammatory pathway via the upregulation of anti-inflammatory genes and downregulation of proinflammatory genes. The transcription of PPAR-*γ* target gene regulation occurs when ligand binds to PPAR-*γ* and PPAR-*γ*-RXR heterodimers formed, then it binds to PPRE of DNA of target gene.

PPAR-*γ* has been demonstrated to be involved in adipogenesis and differentiation, and its involvement in other tissues specifically in central nervous system is rapidly emerging [16, 18–20]. PPAR-*γ* is shown to have a vital role in adipocyte differentiation both in vivo and in vitro [1, 21, 22]. Recent studies demonstrate PPAR-*γ* agonists to prevent inflammation and neuronal death after focal cerebral ischemia in rodents [15, 23–25]. Thiazolidinediones (TZDs) are potent synthetic agonists of PPAR-*γ* shown to induce neuroprotection after cerebral ischemia by blocking inflammation. In a recent study, pioglitazone and rosiglitazone (Figure 1(a)) treatment in SCI in adult rat significantly decreased the lesion size, motor neuron loss, myelin loss, as well as astrogliosis and microglial activation due to SCI [16]. These TZDs significantly enhanced the motor function recovery after SCI. The beneficial and protective lipid-independent effects of TZDs are the anti-inflammatory capacities of PPAR-*γ* [4]. TZDs inhibit the expression of various inflammatory proteins like inducible nitric oxide synthase (iNOS), tumor necrosis factor-*α* (TNF-*α*), and matrix metalloproteinase-9 (MMP-9) in macrophages [26] and are beneficial in disorders such as inflammatory bowel disease [27]. These inflammatory molecules are shown to be neurotoxic in models of neurodegenerative diseases, for example, in ALS [28–30]. Several anti-inflammatory mechanisms have been suggested, including inhibition of nuclear factor kappa B (NF-*κ*B), activator protein-1 (AP-1), in addition to signal transducers and activators of transcription (STAT) transcription factors by PPAR-*γ* [31]. Although nuclear receptors repress target genes in the absence of ligand by recruiting corepressors, the molecular mechanism for transcriptional repression by nuclear receptors in response to the binding of ligands await further research. It is possible that PPAR-*γ* is involved in the reciprocal inhibition of differential transcription systems through limited availability of shared cofactors. Recently, an alternative mechanism suggested that a functionally distinct pool of PPAR-*γ* is susceptible to ligand-dependent sumoylation (covalent attachment of small ubiquitin-like modifier) at lysine 365, leading to recruitment and stabilization of nuclear corepressor (N-CoR) complexes at the promoters of proinflammatory genes thereby repressing them [32].

## 2. PPAR-*γ* AND AMYOTROPHIC LATERAL SCLEROSIS

Amyotrophic lateral sclerosis (ALS) is a devastating fatal neurodegenerative disorder characterized by a loss of upper and lower motor neurons. Oxidative stress, mitochondrial dysfunction, and neuroinflammation have been implicated in ALS pathogenesis (Figure 1(b)). PPARs, in particular PPAR-*γ*, may be a major signaling pathway involved in neuroinflammation in ALS. The activation or inactivation of PPAR-*γ* could provide a viable and promising approach to understand the mechanism of neuroinflammation in ALS (Figure 1(b)). Since neuroinflammatory pathway has become one of the hallmarks of ALS [29, 33, 34], therefore, blockage of neuroinflammation is of great interest because of the potential efficacy in ALS patients. PPAR-*γ* has been identified as a key regulatory factor in the modulation of target genes with PPAR response element (PPRE) in their promoters, including those encoding for inflammation (iNOS, NF-*κ*B, COX-2), oxidative stress, and apoptosis. Synthetic PPAR-*γ* agonists developed in the past 25 years that are used primarily as antidiabetic drugs are suitable candidates and are indispensable to study the role of PPAR-*γ* in ALS which may potentially lead to beneficial effects in ALS patients.

Previous studies have shown the protective effect of PPAR-*γ* agonists in many experimental models such as in experimental autoimmune encephalomyelitis (EAE) [35], cytokine-induced apoptotic cell death of cerebellar granule cells in vitro and in vivo, and against glutamate-induced cell death in mixed cortical neurons and glia cocultures [36, 37]. Additionally, PPAR-*γ* agonists are reported to be neuroprotective in tyrosine hydroxylase positive neurons in substantia nigra when exposed to 1-methyl-4-phenyl-1,2,3,6-tetrahydropyridine (MPTP) [18, 19].

## 3. PIOGLITAZONE IS NEUROPROTECTIVE IN ALS

We have tested the neuroprotective effect of pioglitazone in transgenic G93A SOD1 mouse model of ALS and showed that pioglitazone treatment improved motor performance, delayed weight loss, attenuated motor neuron loss, and significantly increased survival by delaying the onset of ALS [38]. Our results also show that pioglitazone treatment reduced microglial activation and gliosis in the spinal cord as assessed by immunohistochemical staining for CD40 (microglia marker) and GFAP (astrocyte marker), respectively. Furthermore, we showed that pioglitazone treatment reduced iNOS, NF-*κ*B, and 3-nitotyrosine immunoreactivity in the spinal cord of G93A transgenic mice.

Our findings were also confirmed by another study on the effect of pioglitazone treatment in G93A SOD1 transgenic mouse model of ALS [39]. In this study, PPAR-*γ* agonist treatment improved survival, muscle strength, and weight loss in ALS mice. Quantification of motor neuron loss was performed at 90 days of age where approximately 30% of motor neurons were lost in G93A mice spinal cord. Pioglitazone treatment completely prevented this motor neuron loss in the spinal cord of G93A mice. They also showed significant reduction in microglial activation as well as reduction in the expression of COX-2 and iNOS [39].

Further evidence in the modulation of proinflammatory markers by pioglitazone were reported by Schütz et al. which suggests that mRNA levels of two cytokine suppressor genes, suppressor of cytokine signaling 1 and 3 (SOCS-1 and -3), were increased as assessed by semiquantitative RT-PCR [39]. Others have reported similar increase in SOCS-1 and -3 in response to TZDs in microglia and astrocytes in vitro [40]. The increase in SOCS-1 and -3 is implicated with the inhibition of Janus kinase-signal transducer and activator of transcription (JAK-STAT) in inflammatory signal transduction. Other studies using PPAR-*γ* agonists suggest that the mechanism of actions are also by induction of neuroprotective genes such as heat shock proteins [16]. Recently, Xu and Drew demonstrated that PPAR-*γ* agonists suppress cytokines like IL-12 family in EAE, an experimental model of multiple sclerosis, when treated with 15-d-PGJ_2_ and rosiglitazone [35]. These studies provide evidence that PPAR-*γ* agonist responses are originating from activated glial cells in central nervous system. PPAR agonists are shown to modulate microglia and astrocytes in central nervous system diseases as these cells are chronically activated and thought to contribute to neuroinflammation with pathological abnormalities in degenerative diseases [41]. Pioglitazone treatment in G93A mice showed reduction in gliosis which is another experimental evidence that PPAR-*γ* acts on glial cells in CNS [38]. The action of PPAR-*γ* in neuronal cells needs to be studied.

The preliminary reports on the neuroprotective role of PPAR-*γ* agonist in transgenic mouse model of ALS and other experimental animal models could potentially be a foundation for new series of studies to understand the mechanism and molecular details of PPARs and their role in protecting motor neurons from inflammatory damages in ALS (Figure 1(b)). The mechanisms of how PPAR-*γ* agonists induce neuroprotection by blocking neuroinflammation is not fully understood and further information on the molecular details of PPAR-*γ* in neuroinflammatory pathways will provide crucial insights on the role of PPAR-*γ* in ALS and other neurodegenerative diseases.

## 4. MITOCHONDRIAL DYSFUNCTION IN ALS

Mitochondrial compromise in ALS is substantiated by reports of changes in their structure, number, and localization in motor neurons and skeletal muscle, in familial and sporadic ALS patients [42]. Other studies reported the potential involvement of mitochondria in the pathogenesis of ALS as mitochondrial abnormalities were found in proximal axons, anterior horn of ALS spinal cords [43]. Additionally, defects in respiratory chain complexes have been detected in postmortem muscle and spinal cord of ALS patients. Based on the evidence of mitochondrial dysfunction in FALS-SOD1, it is hypothesized that mutant SOD1 may directly damage mitochondrial function and integrity. Several studies have shown that transgenic mice overexpressing human G93A SOD1 that display most of the ALS symptoms and pathologies have mitochondrial dysfunction. More importantly, several studies suggests that mitochondrial abnormalities occur long before disease onset [44]. Kong and Xu found massive mitochondrial degeneration in motor neurons that are on the brink of dying and even vacuolar and swollen mitochondria are found near motor neuron cell debris in G93A mice [45]. These observations suggest that mitochondrial abnormalities may trigger the onset of ALS. Recently, we and others have shown that wild type and mutant SOD1 are found within mitochondrion which was known to be a cytosolic enzyme [46, 47]. How SOD1 is interacting with mitochondria is unclear and it is being actively investigated. The toxic action of mutant SOD1 in and out of mitochondria could be partly explained as follows. (i) Mutant but not wild type SOD1 binds to heat shock proteins causing an inhibition of chaperon activities. Both mutant and wild type SOD1 bind to antiapoptotic protein Bcl-2, on the outer mitochondrial membrane, blocking its antiapoptotic activity [42]. (ii) The presence of mutant SOD1 in the mitochondria leads to formation of SOD1 aggregates, entrapping Bcl-2, blocking protein importation to mitochondria which may trigger neuronal cell death due to mitochondrial dysfunction [42].

Since PGC-1*α* is known to coordinate mitochondrial biogenesis and regulates mitochondrial function, it is possible to predict that PGC-1*α* could play an important role in ALS. Impairment of PGC-1*α* could contribute to mitochondrial dysfunction in ALS. To date, there is no published data on the role of PGC-1*α* or its expression in the transgenic mouse model of ALS or human ALS postmortem tissues. However, there are reports on the altered or impaired expression of genes in ALS that some of them fit in the PGC-1*α* target genes category [29, 48], suggesting that there may be a prominent role for PGC-1*α* translational machinery in ALS.

Since PGC-1*α* is a PPAR-*γ* coactivator, it is possible that PPAR-*γ* agonists may be able to activate PGC-1*α* and also the PGC-1*α* target genes. Like in HD, a reduction of PGC-1*α* and its target genes expression is attributed to mutant huntingtin, similarly mutant SOD1 could impair PGC-1*α* and expression of its target genes in ALS. Whether mutant SOD1 can impair PPAR-*γ* is yet to be determined. Future studies on PGC-1*α* and PPAR-*γ* in ALS patients and transgenic mice will shed some lights on these pathways in disease development.

## 5. PPAR-*γ* AND HUNTINGTON'S DISEASE

Huntington's disease is an autosomal dominant, fatal neurogenetic disease that affects approximately 1 in 10,000 people [49]. The etiology of HD is shown to be the unstable CAG repeat expansion in the huntingtin gene on chromosome 4 resulting in polyglutamine expansion in huntingtin protein. The polyglutamine expansion causes the aggregation of huntingtin protein and formation of neuronal inclusion bodies as reviewed by Ortega et al. [50]. Mitochondrial dysfunction has been implicated in HD since defects in electron transport chain complexes are evident in several tissues from HD patients and transgenic mouse models of HD [51–55]. The mechanisms for this mitochondrial dysfunction are actively been studied and in spite of some new and novel discoveries and hypotheses, it is not fully understood how mitochondrial dysfunction and oxidative stress and expansion of unstable CAG repeats in huntingtin gene cause HD. Recent reports show that mutant huntingtin interferes with transcriptional PPAR-*γ* coactivator-1*α* (PGC-1*α*) causing impairment on its function in HD, suggesting that mutant huntingtin plays a role in the dysregulation of PGC-1*α*-mediated transcription and activity, impairing mitochondrial function, and leading to HD pathogenesis [56–58]. Weydt et al. found that PGC-1*α* target genes (NDUFS3, CYCS, COX6A1, NDUFB5, ACADM, TFAM, and LDHB) had reduced expression in HD patient and mouse striatum [27]. They also found that HD mice brain mitochondria show reduced oxygen consumption rates. An interesting finding was that the PGC-1*α* and uncoupling protein 1 (UCP-1) circuit was found to be disrupted in the brown adipose tissue (BAT) of HD transgenic mice. This was discovered when HD mice challenged with cold. As in mammals, after cold is sensed in the hypothalamus, an increase in sympathetic tone in the periphery ensues. In rodents, BAT is the tissue that responds to cold. In HD and wild type mice challenged with cold, PGC-1*α* expression increased, but in HD mice UCP-1, expression was not upregulated. However, they showed that PGC-1*α* expression is decreased in the striatum of human HD. They also examined the expression of unclear hormone receptors (PPAR-*α*, RXR-*α*) and transcription factors (NRF-1) that known to rely upon PGC-1*α* for target gene activation, these genes were upregulated, suggesting possible compensatory upregulation of PGC-1*α*-dependent transcription factors in human HD caudate. Weydt et al. study proposes that based on the evidence for impaired energy production and/or impaired responses to oxidative stress, evaluation of metabolic processes occurring in nonneuronal tissues in the periphery may yield factors and pathways that contribute to neurodegenerative diseases. Weydt et al. and Cui et al. studies provide further support that the reduction of PGC-1*α* and its target genes in HD striatum are caused by mutant huntingtin. Weydt et al. stated from personal communication with J. Boats and R.E. Hughes that their yeast two-hybrid screen identified that PPAR-*γ* is a huntingtin interactor, and the interaction was validated for its biological significance by demonstrating an effect of PPAR-*γ* dosage upon HD neurodegeneration in the fly eye [27].

Increased levels of iNOS in HD [59], elevated oxidative damage products such as malondialdehyde, 8-hydroxydeoxyguanosine, 3-nitrotyrosine, and hemeoxygenase in areas of degeneration in HD brain, and increased free radical production in animal models, indicate the involvement of oxidative stress in HD [60]. This important pathway has great promise and must be explored in order to understand the role of PPAR-*γ* and to identify new therapeutic targets for HD.

Rosiglitazone (a PPAR-*γ* agonist) that induces sensitization to insulin was tested in R6/2 transgenic mouse model of HD for the treatment of atypical diabetes in these mice [61]. The effect of glibenclamide (a sulfonylurea) that depolarizes pancreatic beta cells by blocking ATP-sensitive potassium channels to induce exocytosis of insulin leading to increase in insulin levels was also tested in R6/2 mice. Chronic treatment with these two drugs, singly or in combination, did not change the course of diabetes or survival, weight loss of R6/2 mice. In their paper, general characteristics of diabetes in R6/2 mouse model of HD were described such as development of glycosuria by the age of 9.3 weeks. They showed that 72% of surviving R6/2 mice tested positive for glycosuria by 14 weeks of age. In this study, they found that R6/2 mice displayed progressively worsening glucose intolerance. It is perplexing that there was no correlation between the ages of onset of glucosuria and the age at death of R6/2 mice and R6/2 mice with glycosuria die at similar age as those R6/2 mice without it. They tested glibenclamide and rosiglitazone in an acute treatment paradigm in R6/2 mice at 6 weeks and 10 weeks of age. Glibenclamide significantly reduced blood glucose concentrations in R6/2 mice and wild type mice just one hour after challenge, while rosiglitazone did not alter postchallenge blood glucose values in older R6/2 mice or wild type mice. However, the chronic daily treatment with rosiglitazone in combination with glibenclamide significantly reduced the fasting blood glucose concentration in all mice at 10 weeks of age [61]. Although the objective of this study was to examine the onset of diabetes and its possible contribution to the mortality and motor impairment of R6/2 mice, it also provided data for the role of PPAR-*γ* in R6/2 mice. A recent study also reported the use of metformin, another antidiabetes drug in R6/2 transgenic mice [62]. Metformin treatment in R6/2 mice had beneficial effect only in males with lower doses (2 mg/mL) which translate to about 300 mg/kg/day, and only increased survivals modestly while the fasting daily glucose levels was not changed. Metformin had no effect in females and higher dose (5 mg/mL) had no effect in males’ survivals while the glucose level was reduced at 12 weeks of age. Metformin has numerous effects on metabolism, including insulin sensitization [63], increased glucose uptake [64], decrease hepatic glucose synthesis [65], activation of AMP-activated protein kinase (AMPK, an enzyme involved in glucose and fatty acid metabolism) [66], and mitochondrial inhibition [67, 68]. Activation of AMPK is associated with mitochondrial proliferation and biogenesis [69]. Rosiglitazone was used as PPAR-*γ* agonist in R6/2 mice, which could be used as the bases to test the role of PPAR-*γ* in HD, glibenclamide and metformin were used to treat atypical diabetes in R6/2 mice. Metformin treatment in R6/2 mice increased brain AMPK phosphorylation [62] although this needs to be confirmed. Activation of AMPK leads to reduction in ATP-consuming processes and facilitate ATP-generating cellular processes which could be an explanation for the metformin effect in R6/2 males. Metformin was also considered to be effective in R6/2 mice because of its ability to sensitize insulin which leads to facilitation of glucose utilization. However, role of metformin in mitochondrial biogenesis in R6/2 mice was not examined. The protective effect of metformin in R6/2 mice could be the synergistic effect from several pathways including regulation of PGC-1*α* activity through its direct activation of AMPA kinase. Although the exact mechanism underlying mitochondrial biogenesis may vary between tissues, emerging data indicate that substantial overlap exists. Metformin does not belong to any class of PPAR-*γ* agonists although it is an antidiabetic for type-2 diabetes and stabilizes the glucose level.

PGC-1*α* has been implicated in mitochondrial biogenesis through its ability to control number of genes such as nuclear respiratory factor-1,-2 (NRF-1,-2), estrogen related receptor *α* (ERR*α*), and mitochondrial transcription factor A (Tfam) [70]. Compounds like resveratrol have been implicated in mitochondrial biogenesis [71]. Resveratrol has been shown to activate sirtuin 1 (SIRT1) and results in PPAR-*γ*-mediated transcriptional repression, inhibition of adipogenesis, enhanced lipolysis, and the release of free fatty acids [72]. Activated SIRT1 leads to deactylation of PGC-1*α* resulting in an activation of PGC-1*α* [73]. By deacetylating PGC-1*α*, SIRT1 represses glycolysis, increase hepatic glucose output, and modulates mitochondrial function and biogenesis [73].

PGC-1*α* is known as master regulator of mitochondrial biogenesis and is shown to modulate a number of metabolically relevant transcription factors that collectively help in mitochondrial biogenesis (for review see [61, 74]). Although PPAR-*γ* agonist treatments in R6/2 failed, it is premature to conclude that there is no role for PPARs in HD. Therefore, further studies in other models of HD are required to examine other PPAR-*γ* agonists. Moreover, the effect of PPAR-*γ* agonists on the expression and activation of PGC-1*α* in cell culture models of HD may provide preliminary data to plan full-scale studies in animal models of HD. The rationale for that is based on the increasing evidence that PGC-1*α* expression which is downregulated in patients with Huntington's disease and in several animal models of this neurodegenerative disorder [70].

Thiazolidiones and rexinoids induce PGC-1*α* gene transcription in brown and white adipocytes [75]. Since PCG-1*α* shown to have roles in gluconeogenesis, fatty acid oxidation, and adaptive thermoregulation, then it can be predicted that PPAR-*γ* agonists could help HD mice to maintain thermoregulatory function when exposed to cold. Based on the studies on PGC-1*α* knockout mice that shown to have neurodegenerative lesions, particularly in striatum, suggest that PGC-1*α* may have an important function in neurons [76]. However, the neurodegenerative lesions in PGC-1*α* knockout mice do not mimic lesions in HD. The role of PPAR-*γ* in ALS, AD, and Parkinson's disease are backed with evidence [19, 20, 38, 39] while the role of PPAR-*γ* in HD lacks critical evidence and needs to be studied further. Future studies in other transgenic mouse model of HD could shed light on the role of PPARs in HD. Considering recent results on thermoregulation and mitochondrial biogenesis impairment in HD, and potential neuroprotective role of PGC-1*α* in HD, PPAR-*γ* desperately seeking further attention and these types of studies could provide essential data on the role of PPAR-*γ* in HD.

It is possible that TZDs are also involved in mitochondrial biogenesis [77, 78]. Studies in patients treated with PPAR-*γ* agonists indicate that the reduction of insulin resistance is resulted from the activation of PPAR-*γ* [78]. PPAR-*γ*'s natural coactivator is PGC-1*α*. TZDs can mimic the effect of PGC-1*α* on PPAR-*γ*. If PGC-1*α* levels reduces or become inactivated by acetylation, then the activity of PPAR-*γ* could be affected.

## 6. FUTURE PERSPECTIVES

In this review, we highlighted the role of PPAR-*γ* in neurodegenerative diseases, in particular in a mouse model of ALS and HD. The utilization of pioglitazone in a mouse model of ALS by two independent studies provides strong indication for the involvement of PPAR-*γ* in ALS. Whether PPAR-*γ* is involved in HD remains to be clarified as one study showed the treatment of R6/2 mice with rosiglitazone, another PPAR-*γ* agonist, had no beneficial effect.

In the future, we will explore the mechanisms by which PPAR-*γ* agonists produce neuroprotection in a mouse model of ALS and test whether PPAR-*γ* has a role in HD. It would be of great interest to determine whether the effect of PPAR-*γ* is powered by glial or neuronal cells or both in these models. It would also be of great interest to determine the effect of PPAR-*γ* agonist on muscles in ALS and HD mouse models. These studies in complement with in vitro cell culture studies are necessary in determining the role of PPAR-*γ* in ALS and HD. Since a thermoregulatory defect exists in HD mouse models (for review see [70]), it would be very informative to test the effect of PPAR-*γ* agonists on HD mouse models for their effect in thermoregulation. The activation of PGC-1*α* in HD mouse models or overexpression of PGC-1*α* in HD mouse models show efficacy in blockage of neuronal death, and lead to improvement in behavioral phenotypes and increase in survival in several HD mouse models. If these are confirmed, then there is bonafide evidence that activation of PGC-1*α* could be a great therapeutic strategy for HD. The lack of report on the role of PGC-1*α* in ALS is a limiting step on the hypothesis that PGC-1*α* could be a target of investigation or therapeutic for ALS. Mitochondria have been implicated in ALS and PGC-1*α* has possible role in mitochondrial biogenesis, therefore, it would be informative to examine mitochondrial abnormalities and PGC-1*α* in ALS. However, since PPAR-*γ* agonist shown to activate PGC-1*α*, therefore, there is an indirect possibility that PGC-1*α* in connection with PPAR-*γ* could play some role in ALS.
